# Plant Root Architectural Traits Mediate a Trade‐Off Between Suppression and Tolerance of Competitors

**DOI:** 10.1002/ece3.72977

**Published:** 2026-01-22

**Authors:** Hugo Salinas, Erik J. Veneklaas, Elizabeth Trevenen, Michael Renton

**Affiliations:** ^1^ School of Biological Sciences University of Western Australia Perth Western Australia Australia

**Keywords:** Donald's ideotype, functional‐structural plant model, plant competition, plant evolution, plant traits, root architecture

## Abstract

Plants' competitive ability involves both suppressing the growth of neighbours (competitive effect) and resisting or tolerating their suppression (competitive response). Competition for below‐ground resources must be related to the ability of plants to acquire these resources, which is mediated by roots and their morphology. However, the role of root architecture in the competitive ability of plants, and in the possible trade‐offs among growth potential, competitive suppression and competition tolerance involved, has not been extensively studied. We used a functional‐structural root model coupled with an evolutionary algorithm to simulate the evolution of root architectures in five scenarios with different plant densities. We asked (1) does selection under different intraspecific competition scenarios result in different root architectures? and (2) do differences in these architectures result in differences in growth potential and competitive ability, that is, competitive effect and response? Our results indicate that as the number of neighbours increases, selection on traits such as branching angles, gravitropism and branching probability results in root architectures that are deeper and sparser, resulting in lower shoot biomass. We also found a difference in competitive ability among architectures, with a trade‐off between resistance to competition on one hand, and competitive effect and maximum productivity (maximum shoot biomass) on the other: there is not a globally optimal strategy. Our findings have implications for management of invasive species, improvement of crop yield and the study of species co‐existence.

## Graphical abstract

We use a modelling approach to simulate the evolution of root architecture under increasing levels of competition. An increase in the number of neighbours results in the evolution of roots that grow deeper and are less dense, causing plants to have lower maximum fitness but increased competition tolerance.

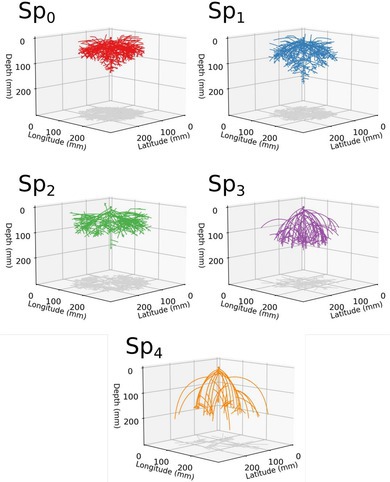

## 1 Introduction

Plants will rarely encounter optimal low‐stress, low‐disturbance or low‐competition conditions, and thus have evolved strategies to withstand non‐optimal conditions during their growth. Grime (1977) identified three extremes of strategies that evolve under different levels of stress (conditions that limit productivity) and disturbance (biomass destruction): the ruderal strategy (low stress and high disturbance intensity), the stress‐tolerant strategy (high stress and low disturbance intensity) and the competitive strategy (low stress and low disturbance intensity). These strategies can be associated with certain traits. For example, plants with the stress‐tolerant strategy tend to have long‐lived, small, evergreen leaves (Grime 1977). Under the competitive strategy, plants maximise fitness in the presence of competitors. As with stress and disturbances, competition intensity varies and different competition intensities should result in the selection of different traits associated with different strategies. Such traits cause differences in competitive ability, which is defined as a species' ability to suppress the growth of neighbouring plants (competitive effect) and to resist suppression by neighbours (competitive response) (Goldberg 1990, 1996; Wang et al. 2010). Studying differences in species' competitive abilities is critical for understanding plant community dynamics, as it is expected that differences in competitive ability eventually lead to species displacement in communities (Hardin 1960; Hart et al. 2018). These differences can also affect yield in agricultural systems (Tokatlidis 2017) and have implications for the management of invasive species (Joshi et al. 2014).

In general, traits that increase a plant's ability to deplete resources, thus making them unavailable to neighbours, increase its competitive effect and traits that allow plants to tolerate or avoid resource depletion by neighbours increase their competitive response (Goldberg 1990). Above‐ground plant traits such as wood density and specific leaf area have been shown to affect the competitive interactions among trees worldwide (Kunstler et al. 2016). The effect of below‐ground traits on competitive ability is less understood (Cahill and Lamb 2007). Roots are the organs that allow plants to forage for below‐ground resources, including water and nutrients (De Kroon et al. 2009; York et al. 2013). How effectively roots access these resources will directly affect plant fitness (Moreau et al. 2022) and likely a plant's competitive ability. Previous theoretical research found that traits important for optimal resource acquisition include root branching angles (Ho et al. 2004), shoot‐root biomass allocation (Iwasa and Roughgarden 1984; Ikegami et al. 2008) and root length per soil depth interval (Jung et al. 2019). Previous work has tended to study the effect of individual root traits on plant performance, but the efficiency of a root system relies on the interaction of multiple traits, which may have synergistic or antagonistic effects (York et al. 2013). Since the whole root system is under natural selection, metrics reflecting properties of the overall root system should be more indicative of plant efficiency and competitive ability than individual traits (Fitter 1987).

Root architecture is the spatial configuration and arrangement of roots within the root system (Fitter 1987; Lynch 1995; Manschadi et al. 2008). It is the result of multiple root traits that are often individually studied and considered important (Cahill and Lamb 2007; Ravenek et al. 2016). Root architecture is determined by a genetic component that is modified by environmental signals (Hodge 2009). Thus, resource limitations should exert a selective pressure on root architectural traits to maximise performance under different conditions. For example, in dry soils, deep roots with greater branching in deeper soil sections are most efficient, whereas in wet soils, having shallower roots with many branches is a better strategy (Draye et al. 2010). Root architecture may therefore be a better indicator of plant efficiency and competitive ability than individual traits. However, its effects on a plant's competitive ability have not been extensively evaluated.

While it is informative to study optimal rooting strategies under specific conditions, it is important to understand how optimal rooting strategies perform under different conditions, as it is unlikely that a strategy is optimal in every condition. Previous research has found that traits that maximise growth when plants are alone are different from those that maximise growth in competition, for example in plant and leaf size of cereals (Hamblin and Donald 1974) and root biomass of wheat (Zhu and Zhang 2013). This relates to what Van Der Bom et al. (2020) called ‘characterising the ecological niche’: understanding and accounting for trade‐offs between root architecture, environmental settings and performance.

Simulation models can help to overcome some of the challenges involved in evaluating root systems to study the relationship between root morphology and performance (Wasson et al. 2012). For example, Javaux et al. (2008) used a 3D model of roots to show that water extraction profiles may not correlate to root density if root radial conductance is large. Renton and Poot (2014) found that specialised root systems with deeper roots evolve in dry shallow soils where deep water is occasionally available. Rangarajan et al. (2018) found that fitness depends on the interaction of root traits such as root angle and basal root whorl number; the fitness landscape of plants is complex. It is also possible to use models to study the relationship between root traits and competition; for example, Rubio et al. (2001) used a root model representing three bean genotypes with shallow, intermediate or deep basal roots, to show that the effect of competition for phosphorus is greater among architectures that explore the same regions of the soil.

Understanding of the relationship between the competitive ability of plants and root system architecture is limited, especially in the context of evolution. To help address this, we aimed to study how root architectures that result from selection under different competition conditions vary in their competitive ability. Specifically, we asked two questions. (1) Does selection under different competition scenarios result in different root architecture traits and root morphology? We hypothesised that scenarios of increasing competitive pressure will result in the selection of root traits that result in deeper root systems that better avoid resource depleted sections in the shallow soil. (2) Can differences in root architecture traits result in differences in growth potential, and competitive effect and response (tendency to suppress growth of neighbours, and resistance to suppression)? We hypothesised that root architecture trait differences would result in different access to sections of the soil and thus different growth potentials. Moreover, architectures that maximise growth potential will be wider and denser and will therefore overlap more with competitors and thus have a higher competitive effect and lower competitive response (be less tolerant of competition).

## 2 Methods

We use the functional structural root model developed by Renton et al. (2025) for use in evolutionary simulations. This model simulates plant roots and their growth as they forage for water in the soil. We use this model as it can represent a very wide array of possible root architectures and the key processes involved in the interactions among growth, root structural development, soil and water, while remaining computationally efficient enough to be used within an evolutionary simulation of many individuals over many generations. An overview of the model is given below, but the reader is referred to Renton et al. (2025) and Renton and Poot (2014) for further details.

### 2.1 Plant Structure

In the model, plants are composed of two biomass compartments (g): below‐ground (root) and above‐ground (shoot and reproductive organs). Root biomass has an explicit three‐dimensional structure, while above‐ground biomass does not. Each root system is composed of branches. Each branch is composed of a set of sequentially joined linear segments, each of the same size (*SegmentLength*). Plants are initialised with a single root branch, with a single root segment heading downward and no shoot biomass. The growth of a plant and the structural development of its root system is determined by a set of architecture parameter values (Table 1) and dynamic interaction with its soil environment. These parameters remain constant within a plant's lifetime but can evolve over generations. The purpose of the model is not to represent any particular species or architectural type, but rather a very wide range of possible architectures, simply through varying the architecture parameter values.

**TABLE 1:** Evolvable architecture parameters and initial value sampling range for the evolutionary algorithm.

Parameter	Parameter description	Parameter limit	Initial value sampling range
rotang	Angle of horizontal rotation from one root lateral to the next (radians)	—	[0, 1.5]
branchang	Angle of vertical rotation from one root lateral to the next (radians)	—	[0, 1.5]
gravitrop	Strength of gravitropism	gravitrop ≥ 0	[0, 0.3]
basezonelength	Length of the base zone (mm)	basezonelength ≥ 0	[5, 30]
basezonep	Probability of branching in base zone	0 ≥ basezonep ≥ 1	[0, 1]
maxorder	Maximum root order	2 ≥ maxorder ≥ 9	[2, 8]
orderweightings	Priority of growth per root order	0 ≥ orderweightings	[0.5, 2]
kshoot1	Controls allocation to shoot biomass per allocable biomass	—	[−5, 5]
kshoot2	Controls allocation to shoot biomass per allocable biomass	—	[−0.1, 0.03]

### 2.2 Model Dynamics

The model simulates the growth of a set of plants over discrete daily time‐steps. At each timestep, the soil water recharges, then plants take up water and then they grow. Plants gather water from the soil, which is divided into voxels. Potential water uptake per root segment is assumed to be constant, so the realised uptake per segment depends on the water content of its soil voxel and the number of segments within the voxel. The total amount of water obtained by a plant is the sum obtained by all its root segments in all the voxels they occupy. Different plants thus compete for water in any voxels where they both have root segments. The total water taken up by the plant is then converted into biomass, a fraction of which is allocated to above‐ground and the rest to below‐ground biomass. This fraction is a function of the obtained biomass and two model parameters (*K*_*shoot1*_ and *K*_*shoot2*_, Table 1). Roots grow in length by adding more segments and by branching with a certain probability; this is controlled by architecture parameters (Table 1). When there is not sufficient allocable biomass to cover the demand of all growing root branches, the priority of the growing branches is determined by their branching order and the *orderweightings* model parameter (Table 1). For further details on how structure, resource uptake and allocation, and growth are represented in the model, and depend on the evolvable architecture parameters (Table 1), see Renton and Poot (2014) and Renton et al. (2025).

In addition to the evolvable architecture parameters (Table 1), the model also depends on model constants, the same values of which were used for all generations within all simulations in this study (Table 2). Roots grow inside a three‐dimensional 20 × 20 × 140 cm soil volume (world) comprised of 10 × 10 × 70 cubic voxels (*x*, *y*, *z*), each with a side length of 20 mm. Each voxel can hold a fraction of its volume in water: they have a moisture content ranging from 0 mm^3^ to 20^3^ × *whc* = 800 mm^3^ of water. The moisture content in the soil is affected by uptake by the plants, diffusion, evaporation and recharge. Diffusion is simulated by moving part of the water content in a voxel to the voxels surrounding it. The diffusible water in a voxel is a fraction *diffuseperday* of its total water content, and 1/6 of this amount moves to each of its six neighbouring voxels. As border conditions, there is no diffusion up from the top layer or down from the bottom layer, while horizontally the border is handled by assuming that edges ‘wrap‐around’ to the opposite edge. Evaporation removes a fraction of the moisture of the top layer of soil voxels. Recharge fills the water content of every voxel to its maximum capacity. Border conditions are avoided by using a wraparound effect on the *x* and *y* dimensions for diffusion and the roots. For further details on how soil water processes are represented in the model see Renton and Poot (2014) and Renton et al. (2025).

**TABLE 2:** Model general constant parameter values.

Constant	Description	Value	Units
waterToBiomass	Biomass obtained per volume of water	0.00001	g mm^−3^
biomassPerVolume	Biomass per unit of root tissue volume	0.001	g mm^−3^
diffuseperday	Proportion of diffusible water per voxel	0.1	
waterTransEffiiciency	Efficiency of water transport from the roots	0.002	
waterUptakePerRootLength	Potential volume of water uptake per mm of root length	10	mm^−3^
maxElongationRate	Maximum daily root elongation	5	mm
SegmentLength	Length of root segment	2	mm
soilvoxelsize	Side length of soil voxel	20	mm
Evaporation rate	Fraction of water content lost to evaporation in the top soil layer	0.5	
whc	Soil water holding capacity, fraction	0.1	

### 2.3 Genotype and Theoretical Species

Each plant has a ‘genotype’, where a genotype means a set of parameter values that affect its growth (Table 1). These parameter values do not change during the growth of the individual plant, but may vary between individuals. Two individual plants with the same genotype will likely have similar root architectures but will vary due to model stochasticity and interactions with their environment. A set of similar genotypes that are the result of a single evolutionary simulation (see below) is called a ‘species’ (Section 2.5).

### 2.4 Competition Scenarios

**FIGURE 1:**
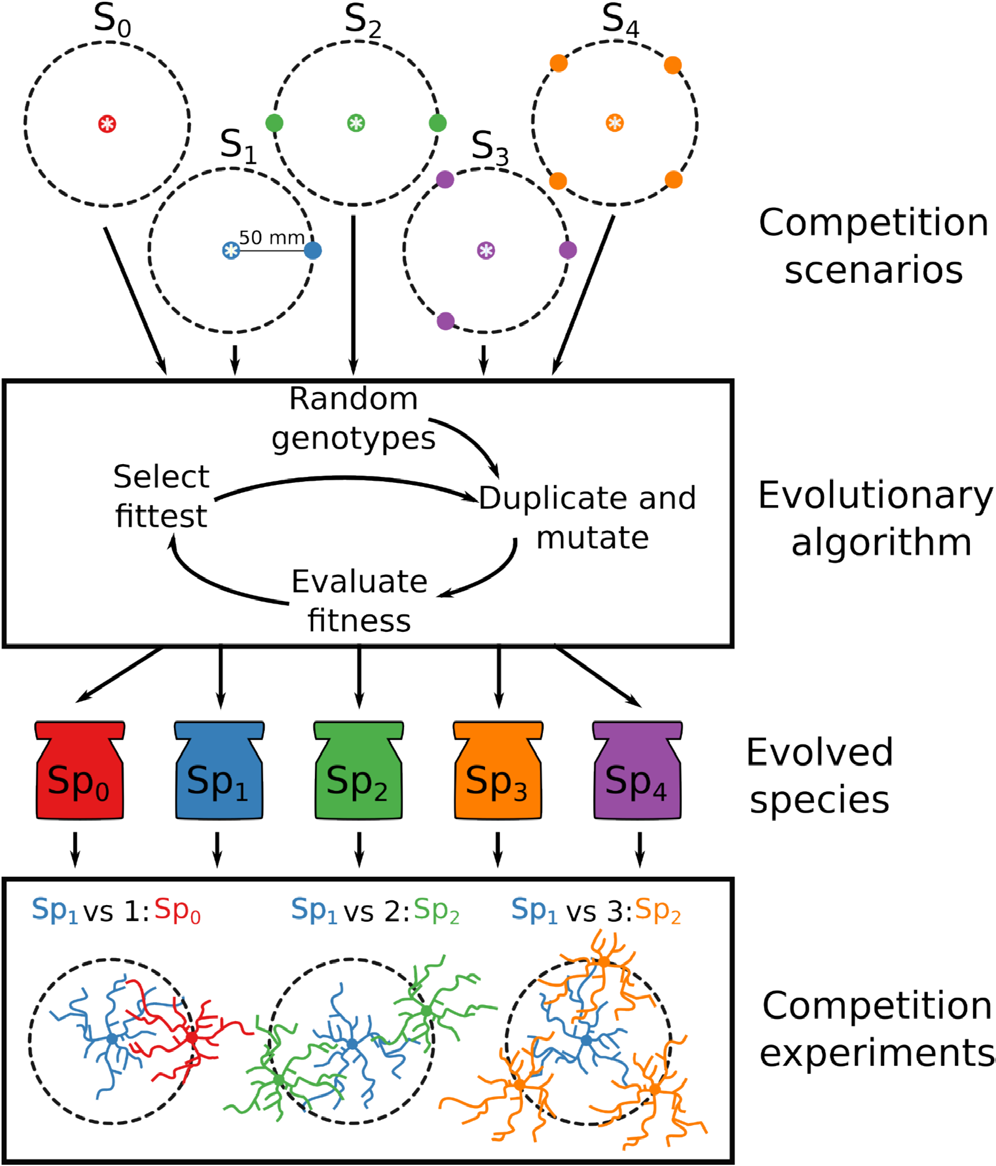
Flowchart of the modelling approach. We use five competition scenarios that differ in the number of competing plants (S_0_–S_4_). Dashed circles indicate the potential location of the neighbours around the target plant which is positioned in the centre of each, indicated with an asterisk. An evolutionary algorithm is used to find sets of genotypes that tend to maximise above‐ground biomass, considering mutations and select above‐ground biomass as the fitness. The resulting genotypes correspond to one of five theoretical species (Sp_0_–Sp_4_). These species are used in a factorial competition experiment considering a target plant in the centre (Sp_0_–Sp_4_), competing with a number (0–4) of plants of the same species (Sp_0_–Sp_4_), The figure shows only three examples of experiments with Sp_1_ as target plant. Colours indicate the competition scenario and species evolved in each; this colour key is used though this study.

We defined five scenarios of varying competition intensity (Figure 1). These considered a target plant developing alone or surrounded by one to four neighbours. The target plant was placed in the middle of the world. The neighbours were placed 50 mm from the target plant (Figure 1). The neighbours had the same genotype; that of the target plant in the evolutionary simulations, but could be different from the target plant in the final competition experiments (see below).

### 2.5 Evolutionary Algorithm

We used an evolutionary algorithm (Ashlock 2006) to find root architecture parameters (Table 1) that result in efficient foraging under each of the five competition scenarios (Figure 1). Above‐ground biomass was used as the measure of fitness since it includes reproductive organs. The evolutionary algorithm consisted of defining a population of *N* = 15 genotypes, each corresponding to a set of parameter values chosen randomly within the sampling ranges in Table 1. This population evolved across 500 generations. At the beginning of each generation, the genotypes were duplicated and added to the population with a random change in all its parameter values (see Table S1). Then, for each of the 30 genotypes in the population (15 original plus 15 copies with mutations) we performed a growth simulation under a competition scenario, with the genotype of any neighbours set to be the same as that of the target plant. The growth simulations ran for 150 time‐steps/days, after which above‐ground biomass of the target plant was recorded. The 15 genotypes with the highest above‐ground biomass of the 30 became the starting population for the next generation. For each competition scenario (S_0_, S_1_, S_2_, S_3_ and S_4_), we performed five independent replicates of this simulated evolution process. We summarise the differences in root parameters across time and scenarios with a principal coordinates analysis on all the root parameter values for a subset of the generations. For each competition scenario, the final populations resulting from the replicate evolutionary simulations were combined, giving a total of 150 genotypes, and the 30 genotypes with the highest final above‐ground biomass were extracted. We thus obtained one group of 30 genotypes for each of the five selection scenarios; we call these genotype groups ‘species’: with species Sp_0_, Sp_1_, Sp_2_, Sp_3_ and Sp_4_ corresponding to scenarios S_0_, S_1_, S_2_, S_3_ and S_4_ respectively. When it was required to simulate the growth of a plant from a given species, one of the 30 genotypes from the corresponding species was randomly assigned.

### 2.6 Root Morphology Comparisons

To compare the morphology of roots across species we performed simulations of plants growing alone. At the end of each 150 time‐step simulation we collected morphological measures: maximum root horizontal length (mm), root depth (mm), root biomass (g), total root length per unit of biomass (mm g^−1^), number of branches per root length (mm^−1^), root density per voxel (g voxel^−1^) and fractal dimension which is a measure of the root complexity (Tatsumi et al. 1989). We used a Principal Components Analysis of the standardised measures to analyse the similarities of the root morphologies corresponding to each species.

### 2.7 Competition Experiments

Finally, we used virtual competition experiments to evaluate the performance (final shoot biomass) of the species when grown alone or in the presence of neighbours. We used a factorial experimental design varying the species of the target plant (Sp_0_ to Sp_4_), the species of the competing neighbours (Sp_0_ to Sp_4_) and the number of neighbours (0 to 4), resulting in (5 × 5 × 4) + 5 = 105 virtual experiments. These experiments thus considered the same competition scenarios as the evolution simulations (see Section 2.2), but with varying species of the target plant and neighbours. 100 replicates of each of these 105 experiments were performed. These experiments again ran for 150 time‐steps. Only the final biomass of the target plant was considered.

## 3 Results

### 3.1 Evolutionary Algorithm

**FIGURE 2:**
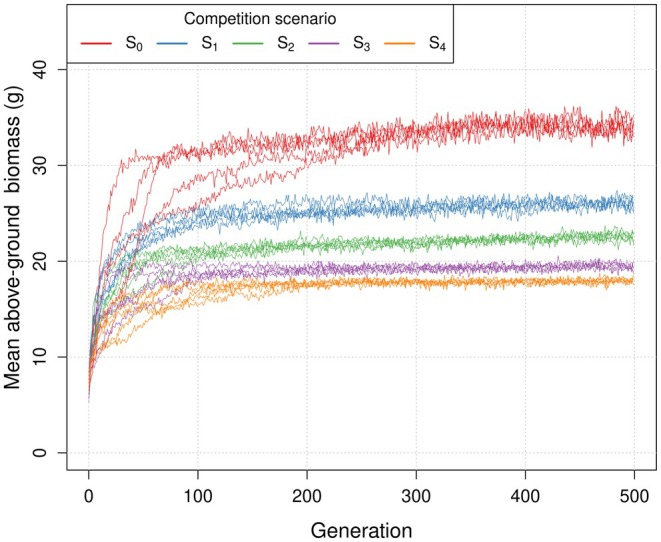
Mean shoot biomass (g) per population of 20 plants across the 500 generations of the evolutionary simulations under the five competition scenarios. Each line corresponds to a replicate run of the evolutionary algorithm. There are five lines per competition scenario, although these are sometimes hard to distinguish.

**FIGURE 3:**
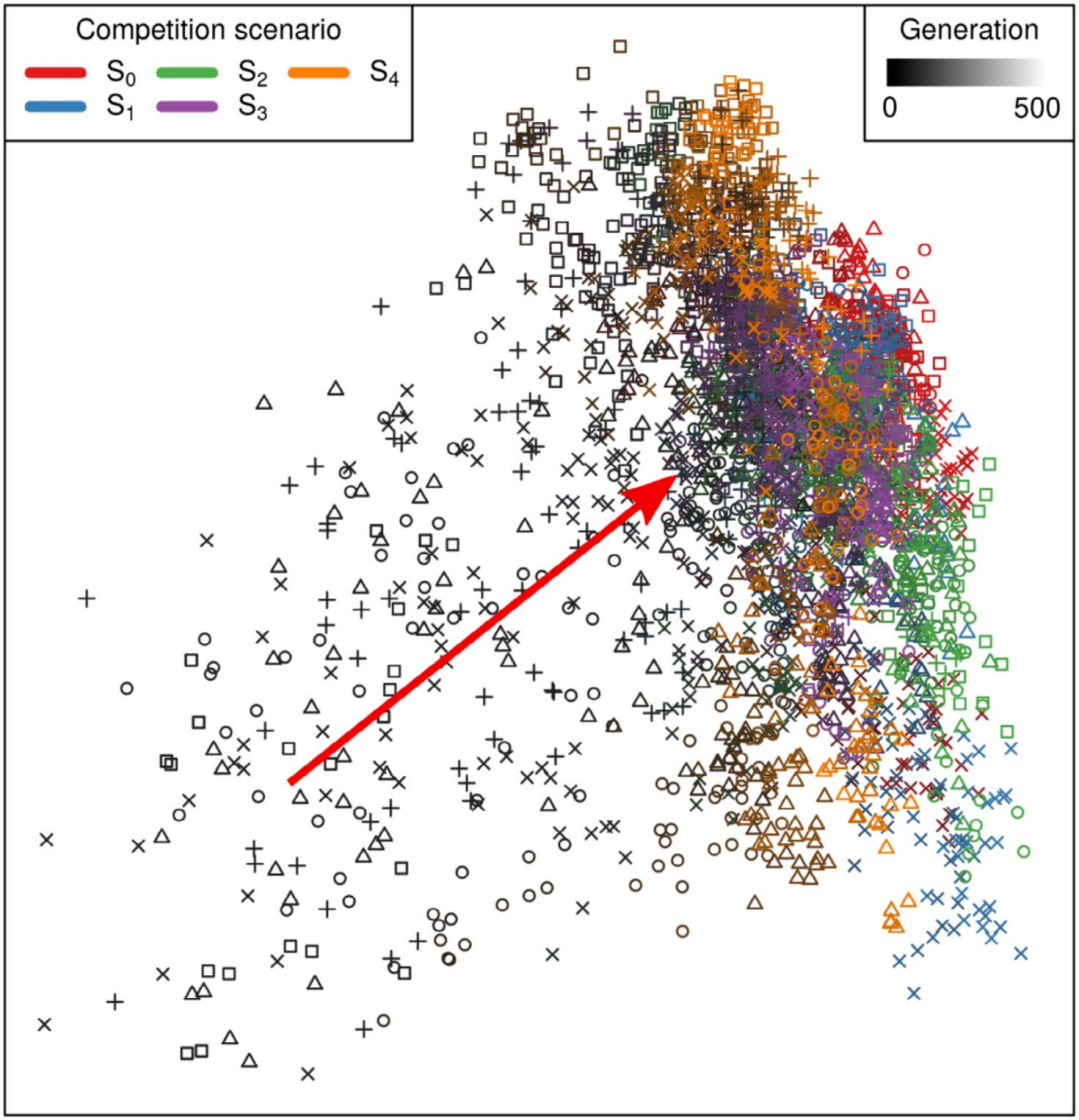
Principal coordinates analysis showing similarities/dissimilarities of the 10 evolvable root parameters across generations of the evolutionary simulation. The data correspond to a subset of 10 genotypes per 15 evenly spaced generations per run. The different colours indicate the competition scenario in which the genotypes evolved. The shade of the colour indicates the generation, ranging from 0 (black) to 500 (colours in legend), with the red arrow indicating the general trajectory of evolution. Symbols indicate to which of the 5 evolutionary runs a genotype belonged.

Mean above‐ground biomass of plants increased over the generations of the evolutionary runs, indicating that the evolutionary simulations were successfully selecting for root architectures that better optimised resource uptake under the different competition scenarios. Different ranges of biomass were reached in different competition scenarios, with greater biomass in scenarios with fewer competitors (Figure 2). The fitness landscapes of the parameter values differed across scenarios and included patterns such as multiple equally optimal peaks (Figure S1A), mostly flat landscapes (Figure S1B), and one clear optimal peak (Figure S1C). The evolvable traits (architecture parameters) also showed a clear trajectory of change over generations towards differing endpoints (red arrow in Figure 3 from black to coloured points). All genotypes were similar in the earliest generations (darker colours in Figure 3), but in later generations genotypes from the same scenario and evolutionary run tended to be more similar to each other than to those in other scenarios and runs, as shown by points with the same shape and colour tending to be closer with increasing generation in Figure 3.

### 3.2 Root Morphology Comparisons

**FIGURE 4:**
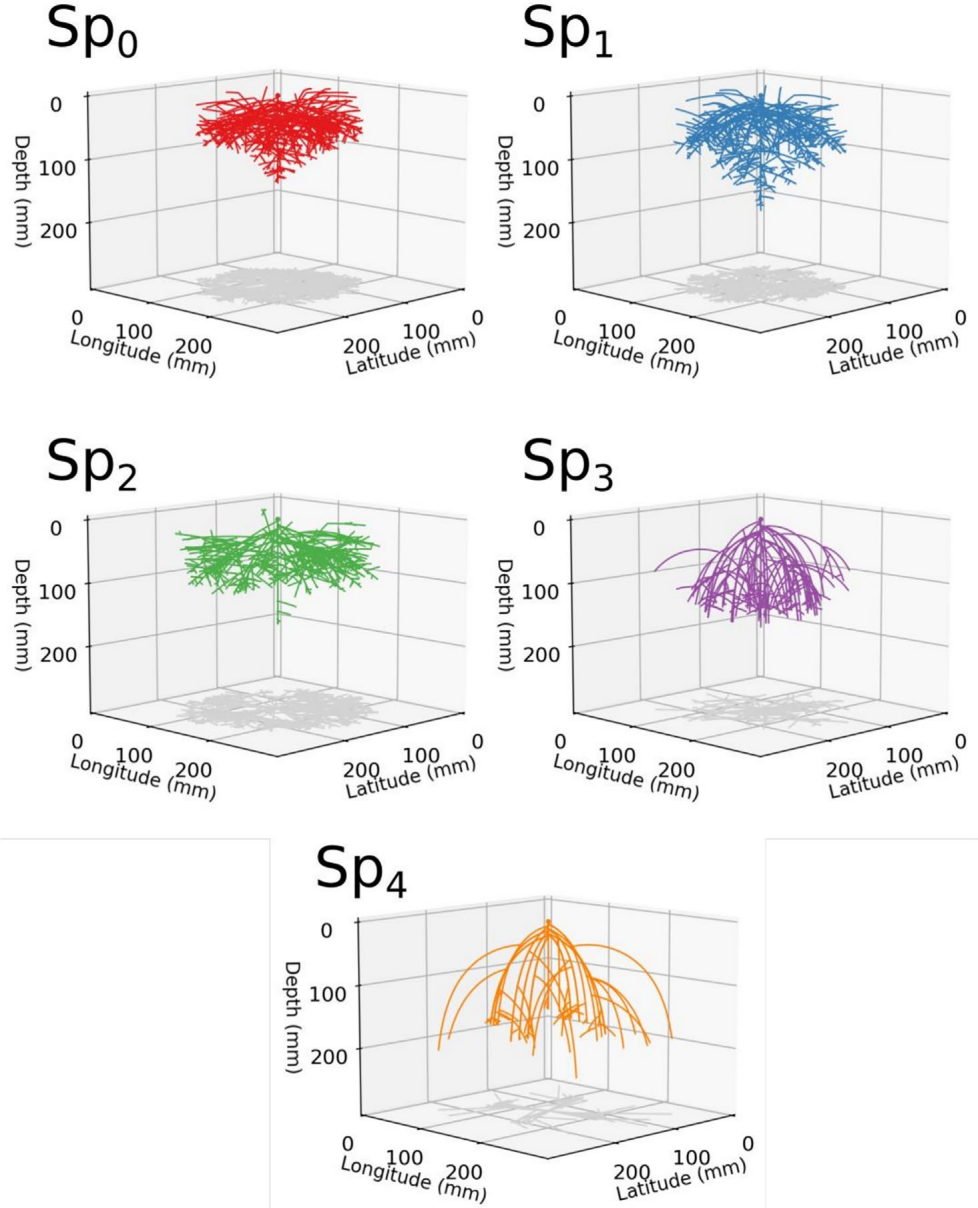
Examples of the root architecture of the five species (Sp_0_–Sp_4_) selected at the end of the evolutionary simulations under the five competition scenarios (S_0_–S_4_). For these examples, a single plant of each species grew alone without competition for 150 days.

**FIGURE 5:**
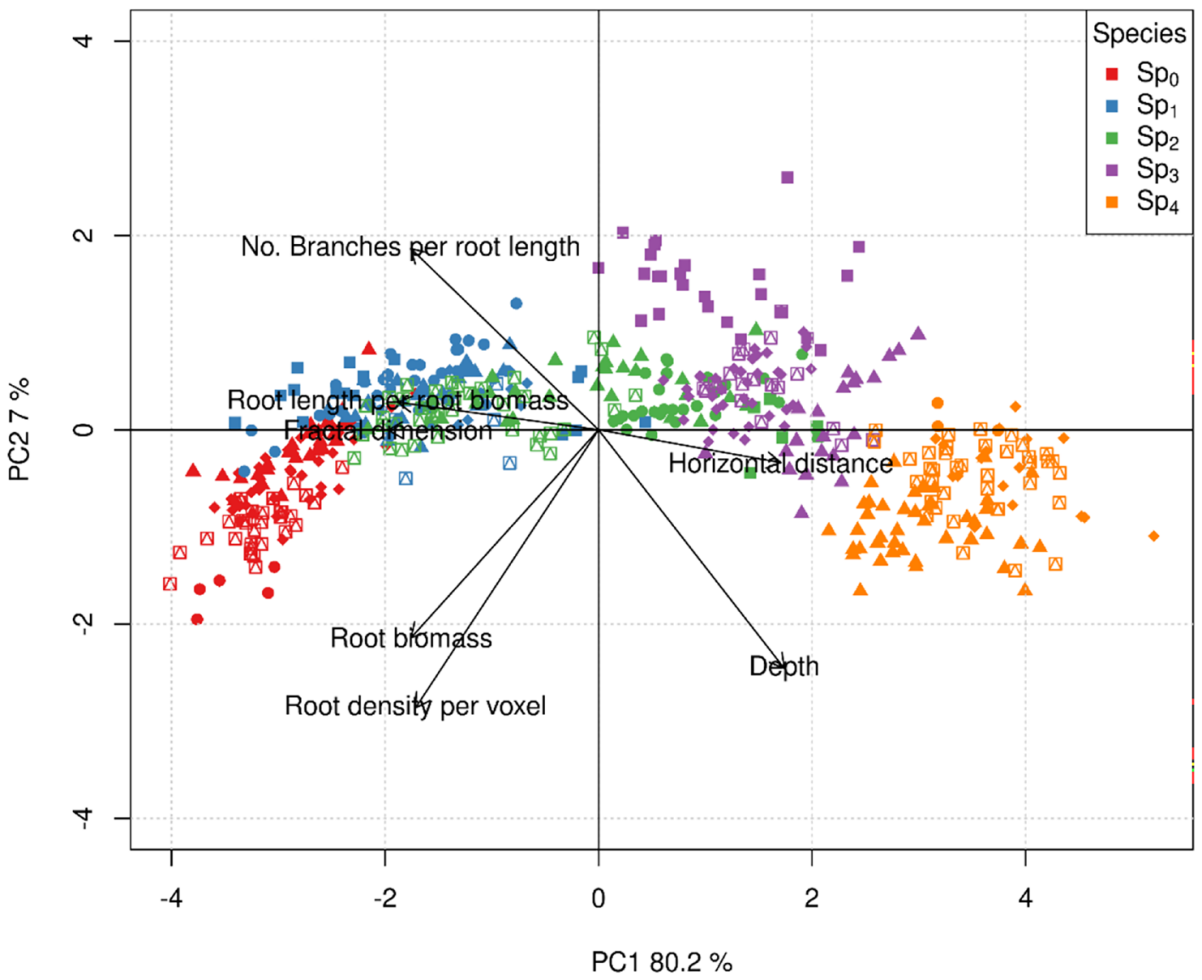
Principal components analysis of emergent root morphological measures of the species evolved at the end of the evolutionary simulations. Each point represents a single individual plant grown alone without competition for 150 days. Colours represent the species of the plant (Sp_0_–Sp_4_); different symbols indicate the five different replicate model runs in which the genotypes were selected. The morphological measures used for the PCA were standardised and are: maximum root horizontal length (mm), root depth (mm), root biomass (g), total root length per unit of biomass (mm g^−1^), number of branches per root length (mm^−1^), root density per voxel (g voxel^−1^) and fractal dimension.

The evolved species varied in their root architectures, with a gradient in root morphology according to the competition scenario in which genotypes were selected, whereas plants of the same species were morphologically similar to each other even across replicate evolutionary runs (Figures 3, 4, 5). Selection under less competition resulted in higher root biomass, root length, fractal dimension, number of branches and lower root depth. Root systems from each of the species clearly differed in the sections of the soil that they occupy; Sp_0_ tends to have more root biomass in the shallow sections of the soil, and close to its base root (Figure 4), while Sp_4_ has sparser deeper roots (Figure 4).

### 3.3 Competition Experiments

**FIGURE 6:**
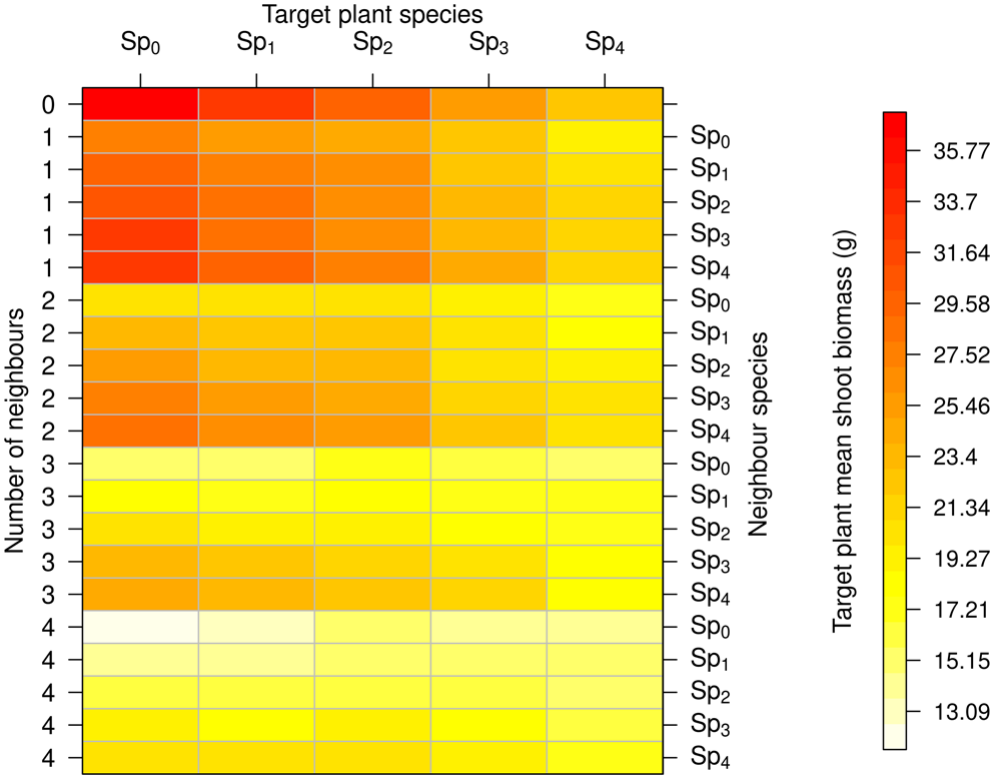
Above‐ground biomass for the competition experiments. The mean of the target plant from 50 repetitions of each experiment is shown. Columns correspond to the species of the target plant, and rows to competition scenario, differing in species and number of neighbours.

**FIGURE 7:**
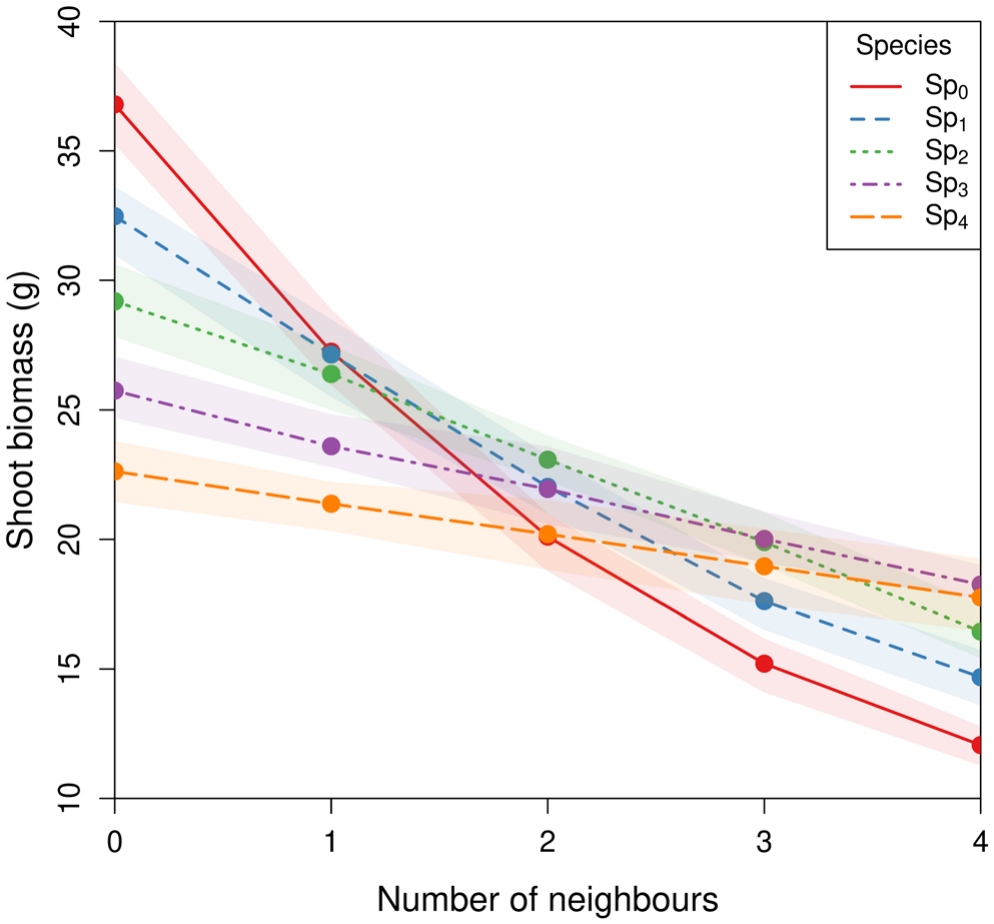
Mean above‐ground biomass for plants of species Sp_0_–Sp_4_ growing with 0–4 intra‐specific neighbours. Lines represent means across 50 repetitions per experiment, and shaded bands are the 25th and 75th quantiles.

The maximum mean above‐ground biomass (36.7 g) was that of plants from Sp_0_ when growing alone, while the lowest (12.0 g) was also from Sp_0_ but under competition with four neighbours from Sp_0_. For each species, plants had maximum above‐ground biomass when they grew without competition (Figure 6). Above‐ground biomass was lower when competing with a higher number of neighbours and when competing with species selected under less competition (such as Sp_0_ and Sp_1_). Intra‐specific competition with four neighbours reduced the biomass of Sp_0_ from 36.7 to 12.0 g on average, while the same level of intra‐specific competition reduced the biomass of Sp_4_ from 22.6 to 16.6 g (Figure 7).

## 4 Discussion

Our results support our first hypothesis that scenarios of increasing competitive pressure will result in the selection of root traits that result in deeper root systems. We found that species that evolved under the same competition scenario converged to similar morphological root traits, even across different replicates of the evolutionary algorithm, and that these differed from plants developed under different competition scenarios (Figures 3, 4, 5). This indicates that there were certain root architectures that performed better in each of the competition scenarios, as indicated by the different fitness landscapes across scenarios (Figure S1). Species evolved strategies to optimise the trade‐off between maximising water extraction whilst minimising investment into root biomass, with these strategies forming a gradient corresponding to the density of neighbours. At the high density extreme of this gradient, represented by Sp_4_, root systems were selected to be less dense and with deeper roots to avoid allocating root biomass to soil sections that would be under high competition (Figures 4 and 5). At the other extreme of this gradient of species, exemplified by Sp_0_, the lack of competition resulted in the selection of a denser and shallower root system, as long deep roots were not needed (Figures 4 and 5).

Our results also support our second hypothesis that root architectures that maximise growth potential will be denser and therefore will overlap more with competitors and thus will have a higher competitive effect and lower competitive response. For all species, target plants had lower shoot biomass when competing with Sp_0_, and higher shoot biomass when competing with Sp_4_ (Figure 6). This indicates that Sp_0_ had a higher competitive effect, that is, a greater ability to suppress the growth of neighbours. This finding is consistent with a review of 72 studies on herbaceous plants (Garbowski et al. 2020), which found that specific root length was correlated with higher competition effect due to a rapid depletion of resources that negatively affected neighbours. This result is also consistent with the fact that, more generally, plant size has been identified as a main indicator of competitive effect (Cahill and Lamb 2007).

Our results also indicate that the species had a different competitive response, or resistance to competition. For example, under the highest competitive pressure (vs. 4 individuals of S_0_), Sp_0_ had an average biomass of 12.0 g, whereas Sp_4_ had an average of 14.4 g (Figure 6). Therefore, Sp_4_ had a greater resistance to competition than Sp_0_. Similarly, in the experiments of Semchenko et al. (2018) on 26 temperate grassland species, plants with deeper roots and lower specific root length were more resistant to competition.

Species also differed in their growth potential (shoot biomass when growing alone), with Sp_0_ having the highest and Sp_4_ the lowest. The selection of traits that increased growth potential (higher shoot biomass in the absence of competition) seems to grant these plants a higher suppression ability. However, the trade‐off for these traits is a reduced performance under competition. A similar trade‐off between strategies that maximise growth potential and resistance to competition has been found in a game‐theoretical analysis by Zhang et al. (1999). Using in situ permanent plots, Tracey and Aarssen (2011) found a trade‐off between minimum and maximum reproductive plant size indicating that the higher the potential size of plants, the more they are vulnerable to competition. Our findings indicate that a potential mechanism underlying this trade‐off is differences in root architecture. This trade‐off indicates that the efficiency of a root architecture depends on the context in which the plants develop: there is not a globally optimal strategy.

The gradient of strategies that evolved reflect two avenues to greater fitness: growth potential (high above‐ground biomass when growing alone) and resistance to competition (limited reduction in above‐ground biomass due to competition). Species S_0_ has the highest growth potential but the lowest resistance to competition. The maximisation of potential growth must involve traits conflicting with those needed to maximise resistance to competition (Schluter et al. 1991). This is to be expected as the peaks of the fitness landscapes already indicate conflicting optimal values for the root traits. Such trade‐offs underpin well‐known ecological strategies (Grime 1977). The pioneering conceptual theory of Donald (1968) suggests that genotypes with a ‘communal type’ will have lower yield in isolation but will have a higher stand yield than plants with a high yield in isolation, suggesting that competition is the mechanism of the trade‐off. Our ‘communal type’ (Sp_4_) has lower shoot biomass than any of the other species when grown alone but performed better under high competition.

The main ecological implication of our findings is that root architectures can cause density‐dependent effects on plant fitness. In a community composed of Sp_0_ and Sp_4_, plants from Sp_0_ attain more biomass when growing under low density and should increase their population size. Then, as high intra‐specific competition heavily affects Sp_0_, there would be a reduction in population size. On the other hand, Sp_4_ would experience a lower average growth rate but could resist higher competition before experiencing population size reduction. General theory suggests that species coexistence will be facilitated if the effect of intraspecific competition is stronger than that of interspecific competition (Chesson 2000; Adler et al. 2018). Our results thus suggest that differences in root architectural traits could favour species coexistence. The results also suggest that some root architectural traits that result in greater competitive resistance could also make species more effective invaders, as suggested by Blank (2010).

Our theoretical approach allowed us to simulate below‐ground competition and to conclude that differences in root architecture can affect the competitive ability of plants. Our species can be ranked across one gradient of strategies: different levels of competition. Similar gradients have been described for other traits. For example, the ‘leaf economic spectrum’ has species with a rapid acquisition and turnover of leaf carbon at one end, and species that efficiently present the carbon they acquire more slowly at the other (Wright et al. 2004; Laughlin et al. 2010). Bergmann et al. (2020) argued that as below‐ground resource uptake may involve collaborations with other organisms, root strategies may be better understood as a multidimensional space. It remains to be studied how root architectures would differ when multiple factors drive their evolution. For example, the effect of root architecture on water uptake likely interacts with physiological processes such as stomatal control, transpiration, hydraulic conductance and water transport through the plant (Tardieu et al. 2017). Architecture traits can also affect mycorrhizal colonisation (Li et al. 2017), which has important consequences for plant fitness. Competition for belowground resources that likely have different spatial distributions and dynamics, for example, water versus nitrogen versus phosphorus should result in the selection of other strategies with other potential trade‐offs (Van Der Bom et al. 2020). Future research should also consider other kinds of plant interactions beyond competition, such as hydraulic redistribution (Neumann and Cardon 2012; Meunier et al. 2018), or plants producing exudates that change soil characteristics, such as water holding capacity or nutrient availability, would likely also change the effect of root interactions (Ahmed et al. 2014; Naveed et al. 2019). Similarly, the interplay between resource heterogeneity (across space and time) and competition remains to be explored, as temporal variation in water recharge would likely result in different strategies (Renton et al. 2025). Theoretical evolutionary functional‐structural approaches such as the one used in this study can be used to study such trade‐offs and interactions in strategies and traits, thus helping to explain empirical observations and develop new hypotheses for further empirical testing.

## Author Contributions

**Hugo Salinas:** conceptualization (equal), data curation (equal), formal analysis (equal), investigation (equal), methodology (equal), project administration (equal), resources (equal), software (equal), visualization (equal), writing – original draft (equal), writing – review and editing (equal). **Erik J. Veneklaas:** investigation (equal), methodology (equal), writing – review and editing (equal). **Elizabeth Trevenen:** conceptualization (equal), methodology (equal), writing – review and editing (equal). **Michael Renton:** conceptualization (equal), formal analysis (equal), funding acquisition (equal), investigation (equal), methodology (equal), project administration (equal), resources (equal), software (equal), supervision (equal), writing – review and editing (equal).

## Conflicts of Interest

The authors declare no conflicts of interest.

## Supporting information


**Data S1:** ece373977‐sup‐0001‐AppendixS1.pdf.

## Data Availability

Code and data that support the findings of this study are available at the Zenodo repository https://doi.org/10.5281/zenodo.18295492.

## References

[ece372977-bib-0001] Adler, P. B. , D. Smull , K. H. Beard , et al. 2018. “Competition and Coexistence in Plant Communities: Intraspecific Competition Is Stronger Than Interspecific Competition.” Ecology Letters 21: 1319–1329. 10.1111/ele.13098. 29938882

[ece372977-bib-0002] Ahmed, M. A. , E. Kroener , M. Holz , M. Zarebanadkouki , and A. Carminati . 2014. “Mucilage Exudation Facilitates Root Water Uptake in Dry Soils.” Functional Plant Biology 41, no. 11: 1129–1137. 10.1071/FP13330. 32481063

[ece372977-bib-0003] Ashlock, D. 2006. Evolutionary Computation for Modeling and Optimization. Springer‐Verlag New York. 10.1007/0-387-31909-3.

[ece372977-bib-0004] Bergmann, J. , A. Weigelt , F. Van Der Plas , et al. 2020. “The Fungal Collaboration Gradient Dominates the Root Economics Space in Plants.” Science Advances 6, no. 27: 1–9. 10.1126/sciadv.aba3756. PMC7458448 32937432

[ece372977-bib-0005] Blank, R. R. 2010. “Intraspecific and Interspecific Pair‐Wise Seedling Competition Between Exotic Annual Grasses and Native Perennials: Plant‐Soil Relationships.” Plant and Soil 326: 331–343. 10.1007/s11104-009-0012-3.

[ece372977-bib-0006] Cahill, J. F. , and E. G. Lamb . 2007. “Interactions Between Root and Shoot Competition and Plant Traits.” HortScience 42, no. 5: 1110–1112. 10.21273/hortsci.42.5.1110.

[ece372977-bib-0007] Chesson, P. 2000. “Mechanisms of Maintenance of Species Diversity.” Annual Review of Ecology and Systematics 31: 343–366. 10.1146/annurev.ecolsys.31.1.343.

[ece372977-bib-0008] De Kroon, H. , E. J. W. Visser , H. Huber , L. Mommer , and M. J. Hutchings . 2009. “A Modular Concept of Plant Foraging Behaviour: The Interplay Between Local Responses and Systemic Control.” Plant, Cell & Environment 32, no. 6: 704–712. 10.1111/j.1365-3040.2009.01936.x. 19183298

[ece372977-bib-0009] Donald, C. M. 1968. “The Breeding of Crop Ideotypes.” Euphytica 17: 385–403. 10.1007/BF00056241.

[ece372977-bib-0010] Draye, X. , Y. Kim , G. Lobet , and M. Javaux . 2010. “Model‐Assisted Integration of Physiological and Environmental Constraints Affecting the Dynamic and Spatial Patterns of Root Water Uptake From Soils.” Journal of Experimental Botany 61: 2145–2155. 10.1093/jxb/erq077. 20453027

[ece372977-bib-0011] Fitter, A. 1987. “An Architectural Approach to the Comparative Ecology of Plant Root Systems.” New Phytologist 106: 61–77. 10.1111/j.1469-8137.1987.tb04683.x.

[ece372977-bib-0012] Garbowski, M. , B. Avera , J. H. Bertram , et al. 2020. “Getting to the Root of Restoration: Considering Root Traits for Improved Restoration Outcomes Under Drought and Competition.” Restoration Ecology 28, no. 6: 1384–1395. 10.1111/rec.13291.

[ece372977-bib-0013] Goldberg, D. E. 1990. “Components of Resource Competition in Plant Communities.” In Perspectives on Plant Competition, edited by J. Grace and D. Tilman , 27–49. Academic Press. 10.1016/b978-0-12-294452-9.50007-2.

[ece372977-bib-0014] Goldberg, D. E. 1996. “Competitive Ability: Definitions, Contingency and Correlated Traits.” Philosophical Transactions of the Royal Society of London. Series B, Biological Sciences 351, no. 1345: 1377–1385. 10.1098/rstb.1996.0121.

[ece372977-bib-0015] Grime, J. P. 1977. “Evidence for the Existence of Three Primary Strategies in Plants and Its Relevance to Ecological and Evolutionary Theory.” American Naturalist 111, no. 982: 1169–1194. 10.1086/283244.

[ece372977-bib-0016] Hamblin, J. , and C. M. Donald . 1974. “The Relationships Between Plant Form, Competitive Ability and Grain Yield in a Barley Cross.” Euphytica 23: 535–542.

[ece372977-bib-0017] Hardin, G. 1960. “The Competitive Exclusion Principle.” Science 131, no. 3409: 1292–1297. 10.1126/science.131.3409.1292. 14399717

[ece372977-bib-0018] Hart, S. P. , R. P. Freckleton , and J. M. Levine . 2018. “How to Quantify Competitive Ability.” Journal of Ecology 106: 1902–1909. 10.1111/1365-2745.12954.

[ece372977-bib-0019] Ho, M. D. , B. C. McCannon , and J. P. Lynch . 2004. “Optimization Modeling of Plant Root Architecture for Water and Phosphorus Acquisition.” Journal of Theoretical Biology 226: 331–340. 10.1016/j.jtbi.2003.09.011. 14643647

[ece372977-bib-0020] Hodge, A. 2009. “Root Decisions.” Plant, Cell & Environment 32: 628–640. 10.1111/j.1365-3040.2008.01891.x. 18811732

[ece372977-bib-0022] Ikegami, M. , D. F. Whigham , and M. J. Werger . 2008. “Optimal Biomass Allocation in Heterogeneous Environments in a Clonal Plant‐Spatial Division of Labor.” Ecological Modelling 213: 156–164. 10.1016/j.ecolmodel.2007.11.016.

[ece372977-bib-0023] Iwasa, Y. , and J. Roughgarden . 1984. “Shoot/Root Balance of Plants: Optimal Growth of a System With Many Vegetative Organs.” Theoretical Population Biology 25, no. 1: 78–105. 10.1016/0040-5809(84)90007-8.

[ece372977-bib-0025] Javaux, M. , T. Schröder , J. Vanderborght , and H. Vereecken . 2008. “Use of a Three‐Dimensional Detailed Modeling Approach for Predicting Root Water Uptake.” Vadose Zone Journal 7: 1079–1088. 10.2136/vzj2007.0115.

[ece372977-bib-0026] Joshi, S. , M. Gruntman , M. Bilton , M. Seifan , and K. Tielbörger . 2014. “A Comprehensive Test of Evolutionarily Increased Competitive Ability in a Highly Invasive Plant Species.” Annals of Botany 114: 1761–1768. 10.1093/aob/mcu199. 25301818 PMC4649698

[ece372977-bib-0027] Jung, Y. , K. Park , K. H. Jensen , W. Kim , and H. Y. Kim . 2019. “A Design Principle of Root Length Distribution of Plants.” Journal of the Royal Society Interface 16: 20190556. 10.1098/rsif.2019.0556. 31795862 PMC6936045

[ece372977-bib-0028] Kunstler, G. , D. Falster , D. A. Coomes , et al. 2016. “Plant Functional Traits Have Globally Consistent Effects on Competition.” Nature 529, no. 7585: 204–207. 10.1038/nature16476. 26700807

[ece372977-bib-0056] Laughlin, D. C. , J. J. Leppert , M. M. Moore , and C. H. Sieg . 2010. “A Multi‐Trait Test of the Leaf‐Height‐Seed Plant Strategy Scheme With 133 Species From a Pine Forest Flora.” Functional Ecology 24, no. 3: 493–501. 10.1111/j.1365-2435.2009.01672.x.

[ece372977-bib-0029] Li, H. , B. Liu , M. L. McCormack , Z. Ma , and D. Guo . 2017. “Diverse Belowground Resource Strategies Underlie Plant Species Coexistence and Spatial Distribution in Three Grasslands Along a Precipitation Gradient.” New Phytologist 216, no. 4: 1140–1150. 10.1111/nph.14710. 28758691

[ece372977-bib-0030] Lynch, J. 1995. “Root Architecture and Plant Productivity.” Plant Physiology 109: 7–13. 10.1104/pp.109.1.7. 12228579 PMC157559

[ece372977-bib-0031] Manschadi, A. M. , G. L. Hammer , J. T. Christopher , and P. DeVoil . 2008. “Genotypic Variation in Seedling Root Architectural Traits and Implications for Drought Adaptation in Wheat (*Triticum aestivum* L.).” Plant and Soil 303: 115–129. 10.1007/s11104-007-9492-1.

[ece372977-bib-0032] Meunier, F. , Y. Rothfuss , T. Bariac , et al. 2018. “Measuring and Modeling Hydraulic Lift of *Lolium multiflorum* Using Stable Water Isotopes.” Vadose Zone Journal 17, no. 1: 1–15. 10.2136/vzj2016.12.0134.

[ece372977-bib-0033] Moreau, D. , H. Busset , A. Matejicek , M. Prudent , and N. Colbach . 2022. “Water Limitation Affects Weed Competitive Ability for Light. A Demonstration Using a Model‐Based Approach Combined With an Automated Watering Platform.” Weed Research 62: 381–392. 10.1111/wre.12554.

[ece372977-bib-0034] Naveed, M. , M. A. Ahmed , P. Benard , et al. 2019. “Surface Tension, Rheology and Hydrophobicity of Rhizodeposits and Seed Mucilage Influence Soil Water Retention and Hysteresis.” Plant and Soil 437, no. 1–2: 65–81. 10.1007/s11104-019-03939-9. 31007286 PMC6447521

[ece372977-bib-0035] Neumann, R. B. , and Z. G. Cardon . 2012. “The Magnitude of Hydraulic Redistribution by Plant Roots: A Review and Synthesis of Empirical and Modeling Studies.” New Phytologist 194, no. 2: 337–352. 10.1111/j.1469-8137.2012.04088.x. 22417121

[ece372977-bib-0036] Rangarajan, H. , J. A. Postma , and J. P. Lynch . 2018. “Co‐Optimization of Axial Root Phenotypes for Nitrogen and Phosphorus Acquisition in Common Bean.” Annals of Botany 122: 485–499. 10.1093/aob/mcy092. 29982363 PMC6110351

[ece372977-bib-0037] Ravenek, J. M. , L. Mommer , E. J. W. Visser , et al. 2016. “Linking Root Traits and Competitive Success in Grassland Species.” Plant and Soil 407, no. 1–2: 39–53. 10.1007/s11104-016-2843-z.

[ece372977-bib-0038] Renton, M. , and P. Poot . 2014. “Simulation of the Evolution of Root Water Foraging Strategies in Dry and Shallow Soils.” Annals of Botany 114: 763–778. 10.1093/aob/mcu018. 24651371 PMC4156118

[ece372977-bib-0039] Renton, M. , H. Salinas , and P. Poot . 2025. “Simulated Evolution of Phenotypic Plasticity in a Functional‐Structural Plant Root Model.” In Silico Plants 7, no. 2: 1–15. 10.1093/insilicoplants/diaf007.

[ece372977-bib-0040] Rubio, G. , T. Walk , Z. Ge , X. Yan , H. Liao , and J. P. Lynch . 2001. “Root Gravitropism and Below‐Ground Competition Among Neighbouring Plants: A Modelling Approach.” Annals of Botany 88: 929–940. 10.1006/anbo.2001.1530.

[ece372977-bib-0041] Schluter, D. , T. D. Price , and L. Rowe . 1991. “Conflicting Selection Pressures and Life History Trade‐Offs.” Proceedings of the Royal Society B: Biological Sciences 246: 11–17. 10.1098/rspb.1991.0118.

[ece372977-bib-0042] Semchenko, M. , A. Lepik , M. Abakumova , and K. Zobel . 2018. “Different Sets of Belowground Traits Predict the Ability of Plant Species to Suppress and Tolerate Their Competitors.” Plant and Soil 424, no. 1–2: 157–169. 10.1007/s11104-017-3282-1.

[ece372977-bib-0043] Tardieu, F. , X. Draye , and M. Javaux . 2017. “Root Water Uptake and Ideotypes of the Root System: Whole‐Plant Controls Matter.” Vadose Zone Journal 16, no. 9: 1–10. 10.2136/vzj2017.05.0107.

[ece372977-bib-0044] Tatsumi, J. , A. Yamauchi , and Y. Kono . 1989. “Fractal Analysis of Plant Root Systems.” Annals of Botany 64: 499–503.

[ece372977-bib-0045] Tokatlidis, I. S. 2017. “Crop Adaptation to Density to Optimise Grain Yield: Breeding Implications.” Euphytica 213: 1–25. 10.1007/s10681-017-1874-8.

[ece372977-bib-0046] Tracey, A. J. , and L. W. Aarssen . 2011. “Competition and Body Size in Plants: The Between‐Species Trade‐Off for Maximum Potential Versus Minimum Reproductive Threshold Size.” Journal of Plant Ecology 4, no. 3: 115–122. 10.1093/jpe/rtr008.

[ece372977-bib-0047] Van Der Bom, F. J. , A. Williams , and M. J. Bell . 2020. “Root Architecture for Improved Resource Capture: Trade‐Offs in Complex Environments.” Journal of Experimental Botany 71: 5752–5763. 10.1093/jxb/eraa324. 32667996

[ece372977-bib-0049] Wang, P. , T. Stieglitz , D. W. Zhou , and J. F. Cahill . 2010. “Are Competitive Effect and Response Two Sides of the Same Coin, or Fundamentally Different?” Functional Ecology 24: 196–207. 10.1111/j.1365-2435.2009.01612.x.

[ece372977-bib-0050] Wasson, A. P. , R. A. Richards , R. Chatrath , et al. 2012. “Traits and Selection Strategies to Improve Root Systems and Water Uptake in Water‐Limited Wheat Crops.” Journal of Experimental Botany 63: 3485–3498. 10.1093/jxb/ers111. 22553286

[ece372977-bib-0055] Wright, I. J. , P. B. Reich , M. Westoby , et al. 2004. “The Worldwide Leaf Economics Spectrum.” Nature 428, no. 6985: 821–827. 10.1038/nature02403. 15103368

[ece372977-bib-0051] York, L. M. , E. A. Nord , and J. P. Lynch . 2013. “Integration of Root Phenes for Soil Resource Acquisition.” Frontiers in Plant Science 4: 1–15. 10.3389/fpls.2013.00355. 24062755 PMC3771073

[ece372977-bib-0053] Zhang, D. Y. , G. J. Sun , and X. H. Jiang . 1999. “Donald's Ideotype and Growth Redundancy: A Game Theoretical Analysis.” Field Crops Research 61: 179–187. 10.1016/S0378-4290(98)00156-7.

[ece372977-bib-0054] Zhu, L. , and D. Y. Zhang . 2013. “Donald's Ideotype and Growth Redundancy: A Pot Experimental Test Using an Old and a Modern Spring Wheat Cultivar.” PLoS One 8: 1–7. 10.1371/journal.pone.0070006. PMC3723726 23936133

