# Supplementary material for: Plant Root Architectural Traits Mediate a Trade‐Off Between Suppression and Tolerance of Competitors
Source: Ecol Evol. 2026 Jan 22;16(1):e72977. doi: 10.1002/ece3.72977 (PMC12827493; doi:10.1002/ece3.72977)
Supplement: Supplementary file 1 — Data S1: ece373977‐sup‐0001‐AppendixS1.pdf. [file ECE3-16-e72977-s001.pdf]

Supplementary material: Plant root architectural traits mediate a trade-off between suppression and tolerance of competitors

Parameter	Initial value range	Value conditions	Mutation conditions
branchsupp	[-0.001, -0.0003]	-	$\times, [0.9, 1.1]$
rotang	[0, 3.13]	-	$+, [-0.1, 0.1]$
branchang	[0, 3.14]	-	$+, [-0.1, 0.1]$
gravitrop	[0, 0.3]	$0 <$ gravitrop	$\times, [0.9, 1.1]$
basezonelength	[5, 30]	$0 <$ basezonelength	$+, [-1, 1]$
basezonep	[0, 1]	$0 <$ basezonep < 1	$+, [-0.1, 0.1]$
maxorder	[2, 8]	$2 <$ maxorder < 8, integer	$+, [-1, 1]$
orderweightings	[0.5 , 2]	$0 <$ orderweightings	$+, [-0.1, 0.1]$
kshoot1	[-5, 5]	-	$+, [-1, 1]$
kshoot2	[-0.1, 0.03]	-	$+, [-0.01, 0.01]$

Table S1: Evolutionary algorithm parameter conditions. Populations in the evolutionary algorithm were initialized with random genotypes with values generated from a uniform distribution within the Initial value range. Across generations mutations changed the parameter values according to the Mutation Conditions by either adding (+) or multiplying ($\times$) the current parameter value by a random number in the range specified

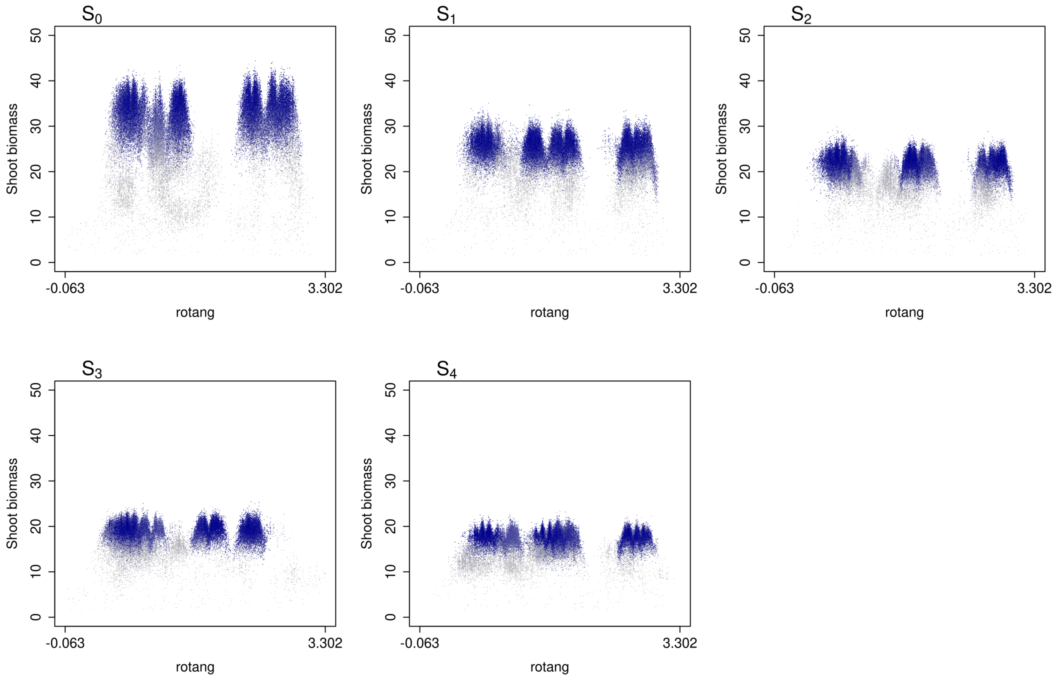

(a) Angle of horizontal rotation

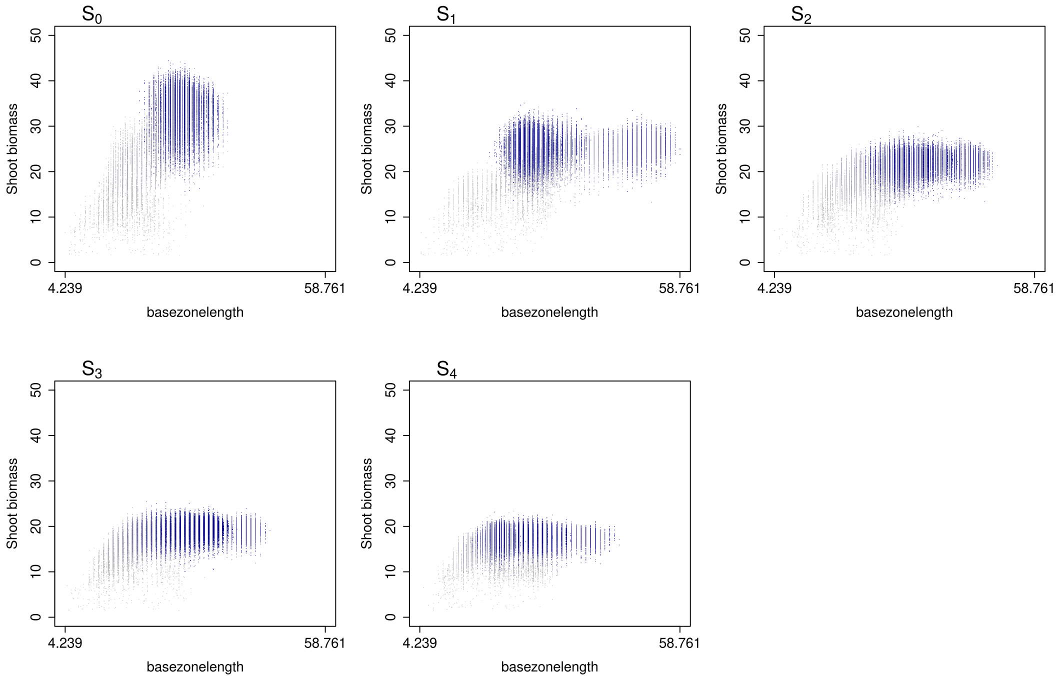

(b) Length of the base zone

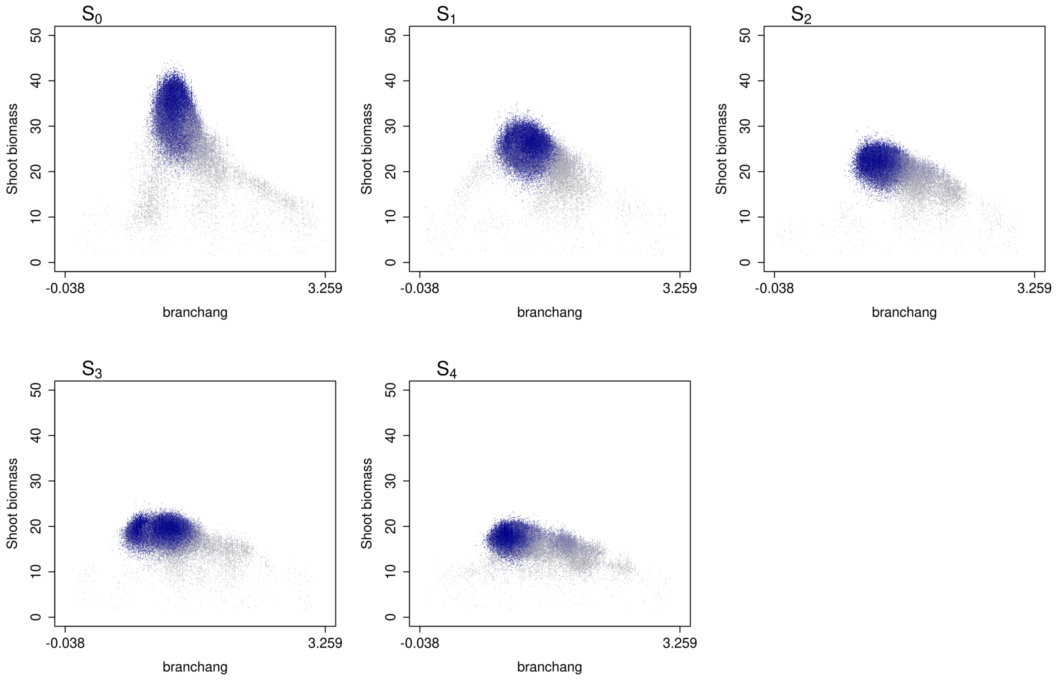

(c) Angle of vertical rotation

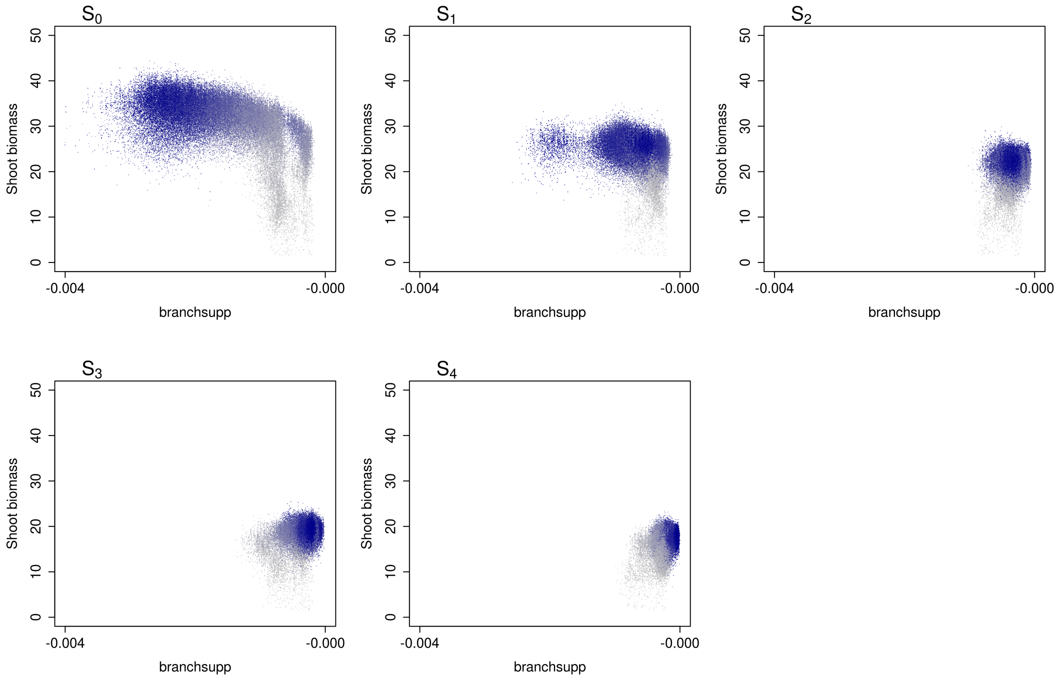

(d) Parameter controlling probability of branching not in the base zone

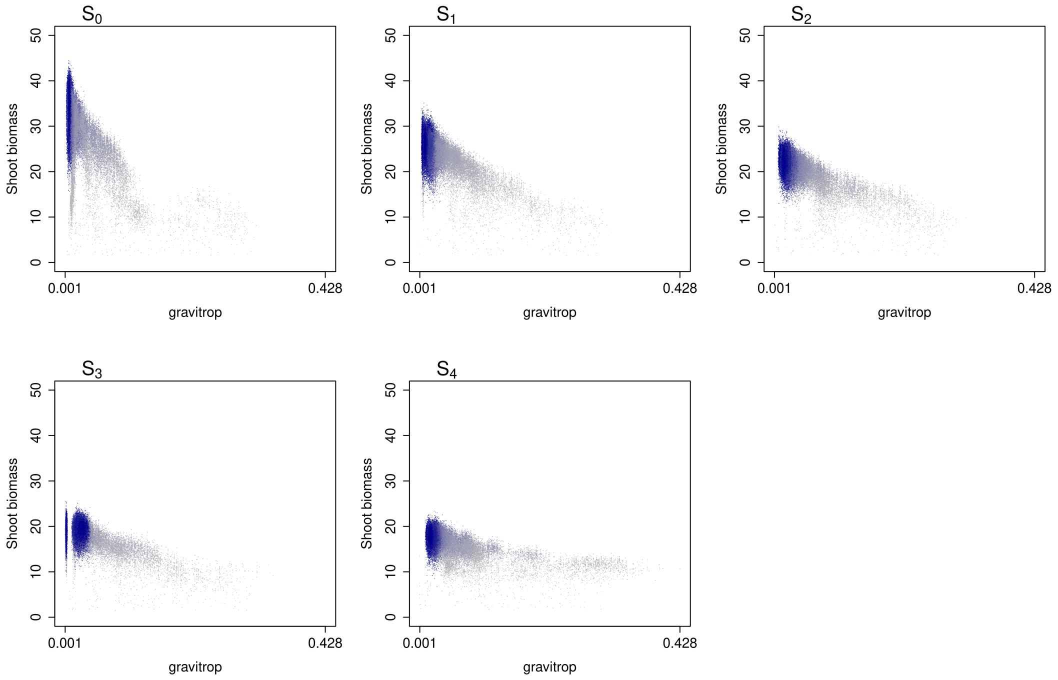

(e) Gravitropism

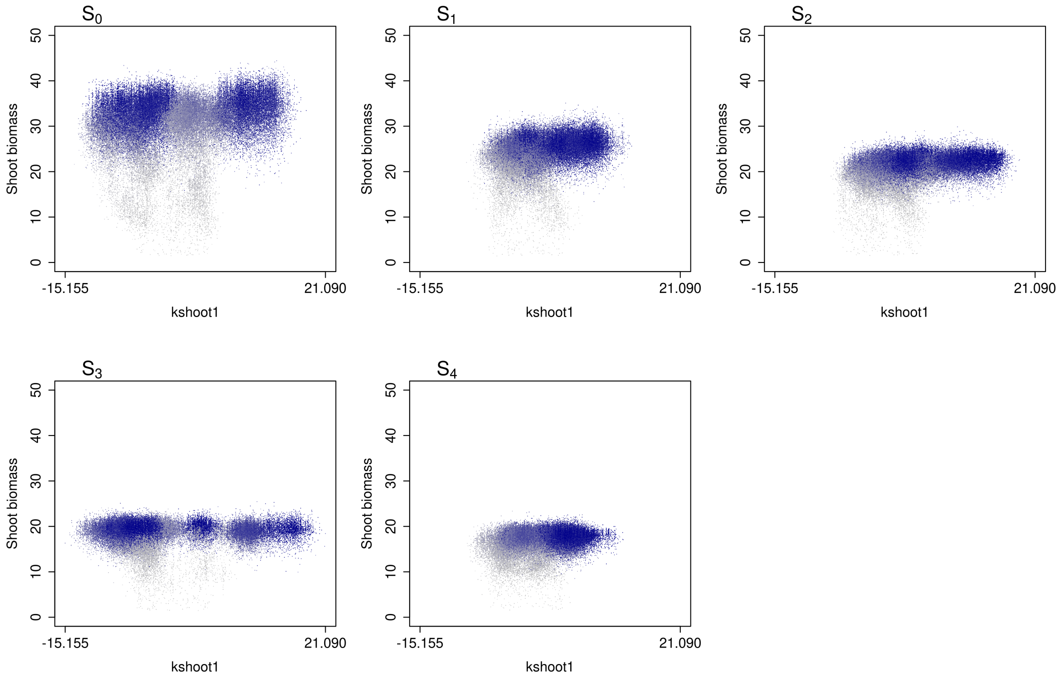

(f) Parameter that controls proportion of biomass allocated to shoot (1)

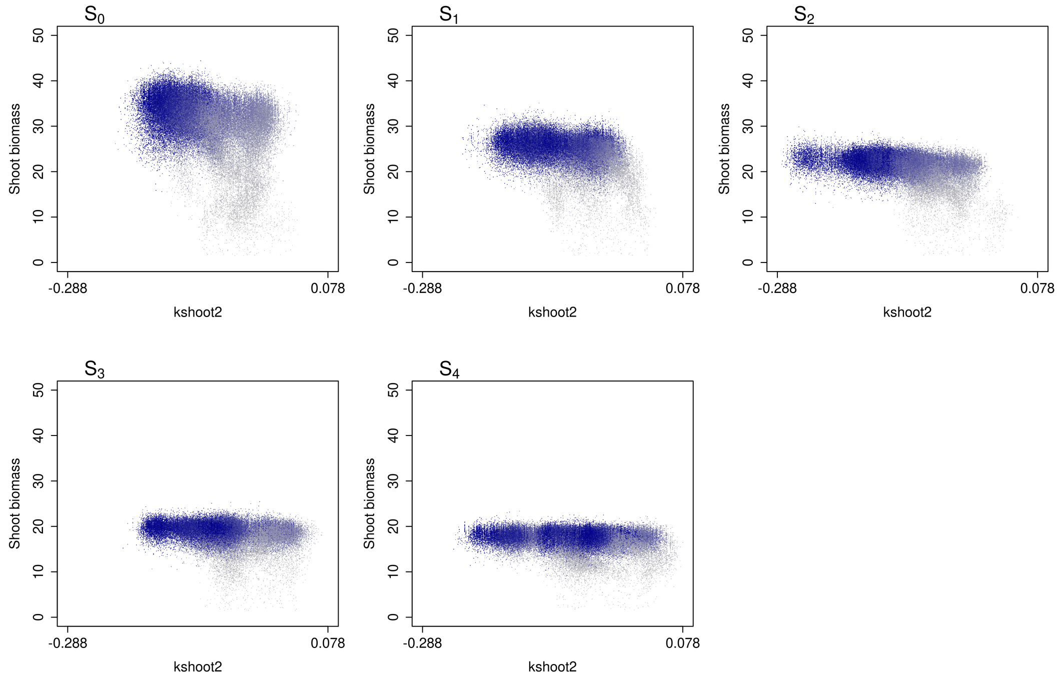

(g) Parameter that controls proportion of biomass allocated to shoot (2)

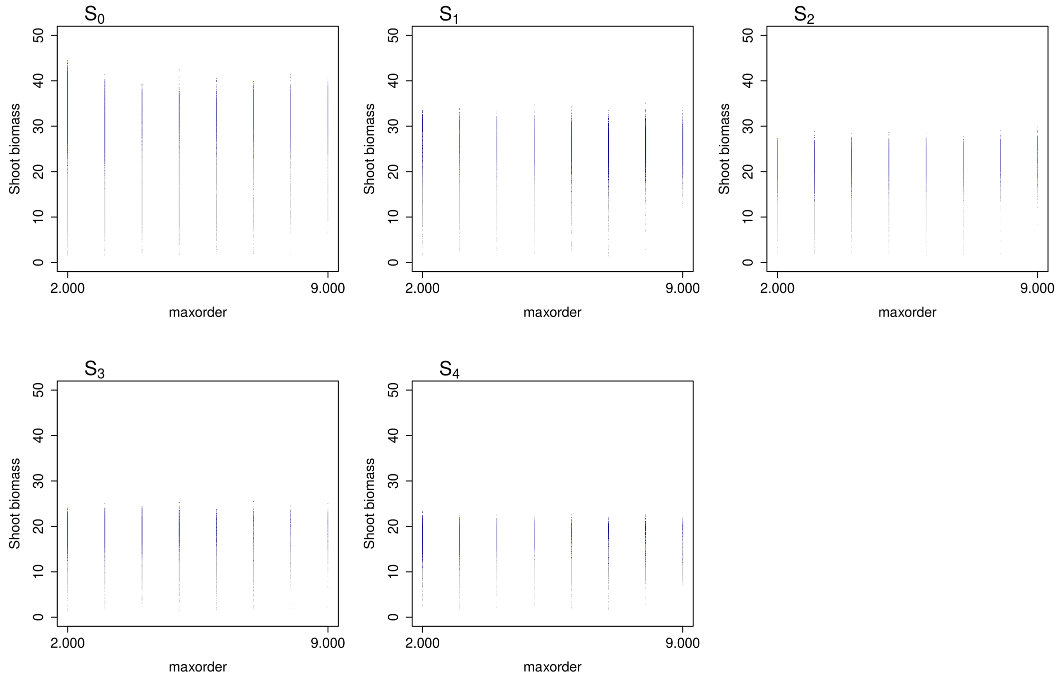

(h) Maximum root order

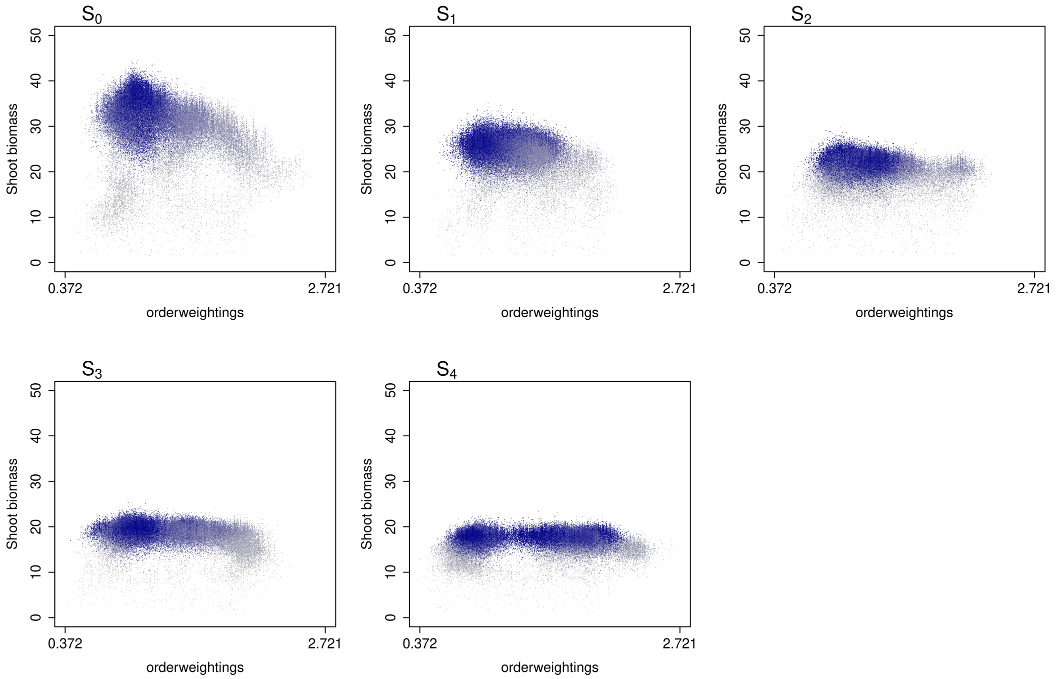

(i) Parameter that controls priority of growth per root order

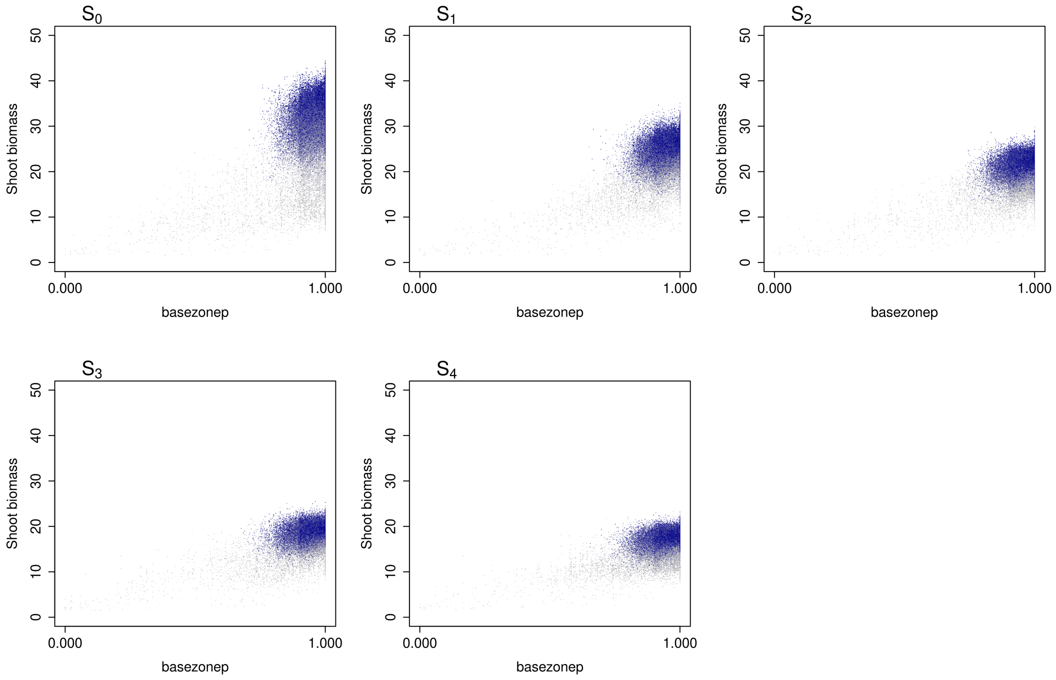

(j) Probability of branching in base zone

Figure S1: Fitness landscape for each parameter values for each competition scenario. Each subplot shows for each of the five competition scenarios (S_0 - S_4) the fitness measure (shoot biomass in grams) per parameter value across all generations and all repetitions of the evolutionary algorithm. Points in gray correspond to earlier generations and darkblue points to generations at the end of the algorithm.
